# Functional Connectivity of Child and Adolescent Attention Deficit Hyperactivity Disorder Patients: Correlation with IQ

**DOI:** 10.3389/fnhum.2016.00565

**Published:** 2016-11-09

**Authors:** Bo-yong Park, Jisu Hong, Seung-Hak Lee, Hyunjin Park

**Affiliations:** ^1^Department of Electronic, Electrical and Computer Engineering, Sungkyunkwan UniversitySuwon, Korea; ^2^School of Electronic and Electrical Engineering, Sungkyunkwan UniversitySuwon, Korea; ^3^Center for Neuroscience Imaging Research (CNIR), Institute for Basic Science, Sungkyunkwan UniversitySuwon, Korea

**Keywords:** attention deficit hyperactivity disorder, connectivity, group ICA, IQ, resting-state fMRI

## Abstract

Attention deficit hyperactivity disorder (ADHD) is a pervasive neuropsychological disorder that affects both children and adolescents. Child and adolescent ADHD patients exhibit different behavioral symptoms such as hyperactivity and impulsivity, but not much connectivity research exists to help explain these differences. We analyzed openly accessible resting-state functional magnetic resonance imaging (rs-fMRI) data on 112 patients (28 child ADHD, 28 adolescent ADHD, 28 child normal control (NC), and 28 adolescent NC). We used group independent component analysis (ICA) and weighted degree values to identify interaction effects of age (child and adolescent) and symptom (ADHD and NC) in brain networks. The frontoparietal network showed significant interaction effects (*p* = 0.0068). The frontoparietal network is known to be related to hyperactive and impulsive behaviors. Intelligence quotient (IQ) is an important factor in ADHD, and we predicted IQ scores using the results of our connectivity analysis. IQ was predicted using degree centrality values of networks with significant interaction effects of age and symptom. Actual and predicted IQ scores demonstrated significant correlation values, with an error of about 10%. Our study might provide imaging biomarkers for future ADHD and intelligence studies.

## Introduction

Attention deficit hyperactivity disorder (ADHD) is a common neurobehavioral disorder that affects both children and adolescents (Schneider et al., 2006; Wolraich et al., 2011; Castellanos and Proal, 2012). ADHD patients show symptoms of inattention, hyperactivity, and impulsivity (American Psychiatric Association, 1994; Conners, 1997). ADHD patients can be divided into three subtypes according to symptoms: inattentive, hyperactive/impulsive, and combined type patients (American Psychiatric Association, 1994; Conners, 1997). Child and adolescent ADHD patients show different behavioral symptoms, particularly hyperactivity and impulsivity (Bresnahan and Barry, 2002; Hurtig et al., 2007; Wehmeier et al., 2010; Wolraich et al., 2011). Adolescent ADHD patients tend to exhibit less hyperactivity than child ADHD patients (Bresnahan and Barry, 2002; Hurtig et al., 2007; Wehmeier et al., 2010; Wolraich et al., 2011). Because indiscriminate behavioral or medication treatments (without considering behavioral differences) might have negative effects on ADHD patients, behavioral differences must be considered to improve ADHD treatments (Barkley et al., 1996; Barnard et al., 2010; Wehmeier et al., 2010; Wolraich et al., 2011).

Many neuroimaging techniques were adopted to explore age related ADHD brain alterations (Bresnahan and Barry, 2002; Frodl and Skokauskas, 2012). Bresnahan and Barry (2002) reported distinct electroencephalogram (EEG) frequency patterns between child/adolescent with ADHD patients and normal controls (NC; Bresnahan and Barry, 2002). Frodl and Skokauskas (2012) reported there were distinct brain volume reduction patterns between child and adult ADHD patients (Frodl and Skokauskas, 2012). Many studies focused on identifying differences between ADHD patients and normal subject and studies focusing specifically on difference between child and adolescent ADHD patients were largely lacking. Here, we focused on identifying group-wise differences between child and adolescent ADHD patients using neuroimaging.

Many neuroimaging studies regarding ADHD have adopted magnetic resonance imaging (MRI) and EEG techniques (Bresnahan and Barry, 2002; Frodl and Skokauskas, 2012). MRI is a useful tool for quantifying brain networks of ADHD patients, as it yields both structural and functional information. Functional MRI (fMRI) measures local brain activity using blood-oxygen-level-dependent (BOLD) signals, and many previous studies adopted fMRI for ADHD research (Booth et al., 2005; Cortese et al., 2012). Raw MRI data are typically processed using standardized software packages (Cox, 1996; Fischl, 2012; Jenkinson et al., 2012). Processed data can be used for connectivity analysis, which treats the whole brain as a complex, connected network (Anwander et al., 2007; He et al., 2007; Bullmore and Sporns, 2009). Connectivity analysis explores how activity in one brain region correlates with activity in another region. Connectivity can be measured with a graph structure using nodes and edges (Bullmore and Sporns, 2009). Nodes are brain regions pre-defined using atlas or functional spatial maps extracted from independent component analysis (ICA) (Tzourio-Mazoyer et al., 2002; Craddock et al., 2012; Smith et al., 2013). ICA is a data driven approach to specify nodes in connectivity analysis and has better sensitivity to detect brain network changes compared to conventional atlas based approaches (Smith et al., 2009, 2013). Edge values reflect correlation between two nodes and can be measured with both weighted and un-weighted approaches (Mumford et al., 2010; Rubinov and Sporns, 2010; Schwarz and McGonigle, 2011; Thomas et al., 2015). The weighted approach does not apply an arbitrary threshold to correlation values and thus considers full range of correlation values (Mumford et al., 2010; Schwarz and McGonigle, 2011; Thomas et al., 2015). The weighted approach is more sensitive than conventional hard threshold approaches (Mumford et al., 2010; Schwarz and McGonigle, 2011). Here, we adopted ICA and weighted approaches to quantify connectivity in ADHD patients.

Intelligence quotient (IQ) tests were designed to assess intelligence, and they are commonly administered using the Wechsler Abbreviated Scale of Intelligence (WASI) (Wechsler, 1999). Full-scale IQ (FSIQ) is considered a general measure of IQ, and is composed of verbal IQ (VIQ) and performance IQ (PIQ) (Wechsler, 1999). Child ADHD patients exhibit different patterns of symptom progression depending on IQ (Cheung et al., 2015). Child ADHD patients with low IQ typically demonstrate persistent ADHD symptoms in adolescence (Cheung et al., 2015). Child ADHD patients with high IQ usually cope better with their symptoms and are more responsive to treatment, and thus tend to grow out of their symptoms in adolescence (Cheung et al., 2015). A previous study reported that childhood IQ is a significant predictor of ADHD symptoms in adolescent and early adulthood (Cheung et al., 2015). As IQ is highly associated with ADHD symptoms, we tried to correlate IQ with neuroimaging analysis stemming from child and adolescent ADHD patients.

Child and adolescent ADHD patients exhibit different behavioral symptoms, particularly hyperactivity and impulsivity (Bresnahan and Barry, 2002; Hurtig et al., 2007; Wehmeier et al., 2010; Wolraich et al., 2011). The behavioral differences are well established but brain network studies to help explain these differences were largely lacking. Thus, this study aims to examine the brain network differences between child and adolescent ADHD patients and investigate whether those network differences were linked with brain networks of hyperactivity/impulsivity. The analysis results of brain networks in ADHD were used to find correlation with IQ, because IQ is a significant predictor of ADHD symptoms in child and adolescent ADHD patients (Cheung et al., 2015).

## Materials and Methods

### Subjects and Imaging Data

This study was carried out in accordance with the recommendations of Institutional Review Board (IRB) of Sungkyunkwan University with written informed consent from all subjects. All subjects gave written informed consent in accordance with the local IRB guidelines. We obtained T1-weighted structure data and resting-state fMRI (rs-fMRI) functional data from the New York University Child Study Center involved in the ADHD-200 database (ADHD-200 Consortium, 2012). The ADHD-200 database is an openly accessible database to researchers. T1-weighted structure data were acquired using a Siemens Magnetom Allegra syngo scanner with the following imaging parameters: repetition time (TR) = 2,530 ms; echo time (TE) = 3.25 ms; field of view (FOV) = 256 mm × 256 mm; and voxel resolution = 1.3 mm × 1.0 mm × 1.3 mm. Rs-fMRI functional data were acquired using the same scanner with the following imaging parameters: scan length = 6 minutes; TR = 2,000 ms; TE = 15 ms; FOV = 240 mm × 240 mm; number of slices = 33; and voxel resolution = 3.0mm × 3.0 mm × 4.0 mm. Fifty subjects with mental disease such as depression, anxiety, social phobia, and dyslexia (except ADHD) were excluded from a total of 222 subjects. Eleven subjects who did not perform IQ and ADHD symptom tests were excluded. The remaining 161 subjects were divided into an ADHD patient group (*n* = 77) and a NC group (*n* = 84). Each group was further divided into child and adolescent groups. Patients under 10 years of age were considered children, and those between 10 and 19 years of age were considered adolescents (Findley, 2003). The ADHD patient group consisted of 32 children and 45 adolescents. The NC group consisted of 28 children and 56 adolescents. We randomly removed a few patients in order to have matched number of subjects (*n* = 28) in each group. We assigned the same number of samples in each group to reduce bias. Finally, 28 child ADHD, adolescent ADHD, child NC, and adolescent NC subjects were considered for the study. We repeated the random removal process three more times and obtained three additional sets of four comparison groups. All four sets of data were analyzed to check the reproducibility of this study. Results of one representative set were reported in the main text and those of the remaining three sets were reported in the Supplementary Material. Our main objective of the study is to identify group differences between child and adolescent ADHD patients. Age matched NC group was necessary so that we could remove effects of the normal aging. If age related difference existed in both NC and ADHD groups, we would not treat it as relevant to aging in ADHD. We considered age related difference relevant only if it existed in ADHD group and not in NC group. Comparison of sex ratio, ADHD scores, and ADHD subtype ratio did not yield significant differences (*p* > 0.05) between the child and adolescent ADHD groups (**Table 1**). There were more boys than girls in ADHD group and it is a natural condition as boys make up larger portion of ADHD patients than girls (Lorberboym et al., 2004; Blum and Chen, 2008; Leirbakk, 2015). Comparison of sex ratio did not yield significant differences (*p* > 0.05) between the child and adolescent NC groups (**Table 1**). Detailed participant information is given in **Table 1**. IQ was measured using the WASI (Wechsler, 1999), and scores related to ADHD symptom were measured using Conner’s Parent Rating Scale Revised, Long Version (CPRS-LV) (Conners, 1997).

**Table 1:** Demographic data of child and adolescent subjects in the attention deficit hyperactivity disorder (ADHD) and normal control (NC) groups (means and standard deviations are reported).

ADHD group	Child (*n* = 28)	Adolescent (*n* = 28)	*p*-value
Gender (male : female)	22:6	22:6	^∗^1
Age (years)	8.59 (0.76)	12.31 (1.80)	<0.001
IA score	71.82 (8.74)	72.07 (9.60)	0.9192
HI score	68.21 (11.89)	71.75 (11.97)	0.2725
C score	72.14 (8.10)	74.39 (10.06)	0.3607
Subtype (IA : HI : C)	7:0:21	7:1:20	^∗^0.9372
FSIQ	110.11 (13.70)	105.32 (14.35)	0.2073
VIQ	110.79 (12.70)	106.29 (14.15)	0.2158
PIQ	106.54 (14.99)	102.89 (14.51)	0.3596
**NC group**	**Child (*n* = 28)**	**Adolescent (*n* = 28)**	***p*-value**
Gender (male : female)	14:14	14:14	^∗^1
Age (years)	8.50 (0.78)	13.68 (2.31)	<0.001
FSIQ	109.04 (12.67)	111.75 (13.52)	0.4417
VIQ	111.46 (14.74)	111.96 (13.44)	0.8950
PIQ	104.61 (12.58)	109.00 (13.00)	0.2042

*^∗^Chi-squared test.*

*IA, inattentive; HI, hyperactive/impulsive; C, combined; FSIQ, full scale intelligence quotient; VIQ, verbal intelligence quotient; PIQ, performance intelligence quotient.*

### Image Preprocessing

T1-weighted structure data were preprocessed using the AFNI software (Cox, 1996), and skull tissue was removed using 3dSkullStrip. Magnetic field bias was corrected using 3dUnifize. All rs-fMRI data were further processed using the FSL software (Jenkinson et al., 2012). The first six MRI volumes were removed to adjust for hemodynamic response. Head motion was corrected using MCFLIRT, and slice timing correction was performed using SLICETIMER. Spatial smoothing with a full width at half maximum (FWHM) of 6 mm was applied. Intensity normalization with a value of 10,000 was applied to the entirety of the time series data. A high-pass filter with a cutoff of 100 s was applied. Functional images were registered onto the preprocessed T1-weighted structure images and subsequently registered to the Montreal Neurological Institute (MNI) standard space.

### Group ICA

All subjects’ preprocessed functional data were temporally concatenated and fed into the FSL MELODIC software (Beckmann et al., 2005). The group ICA approach automatically generated spatially independent maps, termed independent components (ICs) (Smith et al., 2014). The generated ICs were compared with known resting state networks (RSNs) for standardized interpretation (Smith et al., 2009). Cross correlation between ICs and RSNs was calculated with a threshold of 0.45, and only functionally interpretable ICs were kept for further analysis. Functionally interpretable ICs were used as regressors to estimate participant-specific time series (Filippini et al., 2009).

### Network Construction

Connectivity information was assessed with a graph structure using nodes and edges (Bullmore and Sporns, 2009; Rubinov and Sporns, 2010). We adopted a weighted and undirectional network model. Functionally interpretable ICs were represented as nodes. Correlation values of the time series between two different nodes were represented as edges. Edge values were entered into the matrix as elements, and the matrix is referred to as the correlation matrix. The conventional hard thresholding approach aggressively removes the edge weights. We applied soft thresholding to avoid binarizing the correlation matrix using the following formula: 
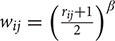
, where *r*_ij_ represents the edge value between the nodes *i* and *j* (Mumford et al., 2010; Schwarz and McGonigle, 2011). The β value was set to 12 in order to conform to the signed network model (Mumford et al., 2010). The correlation matrix was then *z*-transformed using Fisher’s *r*-to-*z* transformation. Network construction was performed using MATLAB (Mathworks Inc., USA).

### Connectivity Analysis

We adopted degree centrality that is a simple and sensitive local connectivity measure (Rubinov and Sporns, 2010). It is defined as the sum of all edge weights connected to a given node (Rubinov and Sporns, 2010). It is one of the most fundamental network measures. Degree centrality could be used to compute clustering coefficient, which is a network related property (Bullmore and Sporns, 2009; Rubinov and Sporns, 2010). A node with high degree centrality could be considered as a hub node which plays an important role in the overall brain network (Bullmore and Sporns, 2009; Rubinov and Sporns, 2010). We adopted two-way analysis of variance (ANOVA) approach to identify brain networks that show significant interaction effects of age (child and adolescent) and symptom (ADHD and NC) (Fujikoshi, 1993). Degree centrality values of each brain network were the dependent variable and age, symptom, and the interaction terms were the independent variables. Age was categorical (i.e., child or adolescent) and symptom was also categorical (i.e., ADHD or NC). We considered the brain networks with significant interaction effects as those affected by both age and symptom. Connectivity analysis was performed using MATLAB (Mathworks Inc., USA).

### Correlation with IQ

Connectivity findings were further analyzed with IQ. Correlation analysis between degree centrality values of identified brain networks and FSIQ, VIQ, and PIQ was performed. Each identified brain network and IQ scores were correlated using a general linear model, IQ = α + β ∗ degree centrality, where α is a constant and β is the estimated coefficient. The significance of the correlation was quantified with *r*- and *p*-value statistics. *P*-values were corrected using the Holm-Bonferroni method (Holm, 1979). The correlation procedures were performed using MATLAB (Mathworks Inc., USA).

### IQ Prediction

A simple linear model used in the correlation analysis was used to predict IQ scores using each brain network. The prediction procedure was performed with a leave-one-out cross validation approach. One subject was used as the test set, and the remaining 55 subjects were used as the training set. A linear equation was generated from the training set and was applied to predict the IQ scores of the test set. The linear model was built from 55 subjects and the model was applied to predict the IQ score of the remaining test case. The remaining test case already has IQ scores available and thus we could compare predicted IQ and actual IQ. The process was repeated 56 times each time choosing a different test case. We computed 56 predicted IQ scores and they were compared with actual IQ scores. The percent error was calculated by dividing the absolute error between actual and predicted IQ scores by actual IQ scores. The mean percent error was reported. The significance of prediction was quantified with *r*- and *p*-value statistics, root mean squared (RMS) values and percent-error. The prediction procedures were performed using MATLAB (Mathworks Inc., USA).

### Statistical Analysis

Interaction effects of age and symptom were assessed using two-way ANOVA approach (Fujikoshi, 1993). Brain networks with significant (*p* < 0.05) interaction effects were regarded as significant networks affected by both age and symptom. The quality of correlation between degree centrality values of each brain network and IQ scores was quantified using *r*- and *p*-value statistics. We applied the Holm-Bonferroni method to obtain corrected *p*-values (Holm, 1979). The quality of the IQ prediction was quantified using *r*- and *p*-value statistics, RMS values and percent-error. All statistical analyses were performed using MATLAB (Mathworks Inc., USA).

## Results

### Spatial Maps from Group ICA

**FIGURE 1:**
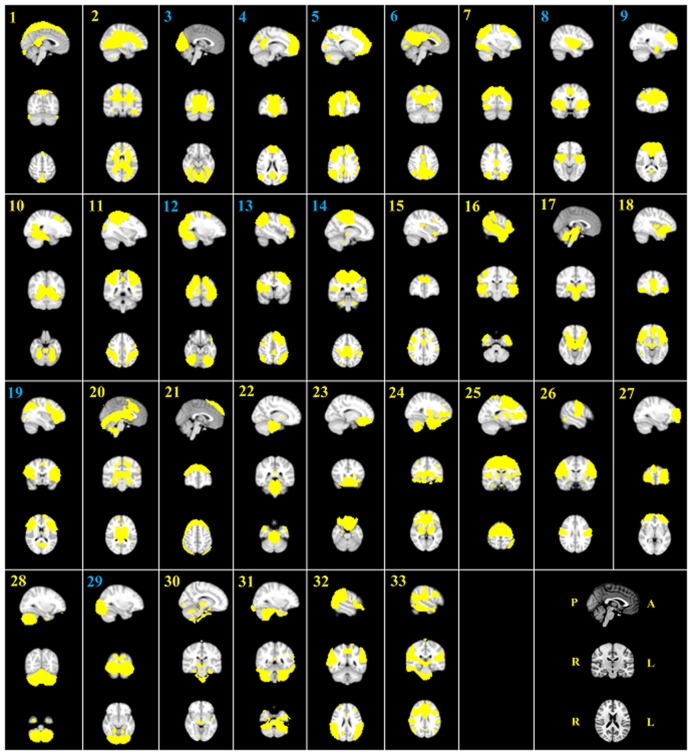
**The 33 automatically generated ICs by group ICA.** All ICs were threshold at *z*-statistic images with *p* > 0.5. The indices of eleven functionally interpretable ICs were reported in blue. P, posterior; A, anterior; R, right; L, left

The group ICA approach automatically generated 33 ICs (**Figure 1**). Generated ICs were compared with known RSNs, and 11 functionally interpretable ICs remained (**Table 2**) (Smith et al., 2009). RSNs 1, 2, and 3 (ICs 3, 29, and 12, respectively) correspond to a visual network consisting of bilateral calcarine, cuneus, lingual gyrus, and superior, middle and inferior occipital gyri. RSN 4 (ICs 4 and 6) corresponds to a default mode network consisting of bilateral medial orbitofrontal gyrus, posterior cingulate cortex and cuneus. RSN 5 corresponds to a cerebellum network. None of the ICs showed significant correlation with RSN 5. RSN 6 (ICs 14) corresponds to a sensorimotor network of bilateral paracentral lobule. RSN 7 (IC 8) corresponds to an auditory network of bilateral Rolandic operculum, insula, putamen, pallidum, and Heschl’s gyrus. RSN 8 (IC 9) corresponds to an executive control network of bilateral superior medial frontal gyrus and anterior cingulate cortex. RSNs 9 and 10 (ICs 5, 13, and 19) correspond to a frontoparieteal network of bilateral superior, middle, and inferior frontal gyri, inferior parietal gyrus, and angular gyrus.

**Table 2:** Functionally interpretable independent components (ICs) and resting state networks (RSNs).

RSNs	ICs	*r*-value	Network	Region
1	3	0.82	Visual	Calcarine, Cuneus, Lingual gyrus, Superior occipital gyrus
2	29	0.82	Visual	Inferior occipital gyrus
3	12	0.69	Visual	Superior, middle, and inferior occipital gyri
4	4, 6	0.47, 0.65	Default mode	Medial orbitofrontal gyrus, Posterior cingulate cortex, Cuneus
5	-	-	Cerebellum	-
6	14	0.46	Sensorimotor	Paracentral lobule
7	8	0.65	Auditory	Rolandic operculum, Insula, Putamen, Pallidum, Heschl’s gyrus
8	9	0.66	Executive control	Superior medial frontal gyrus, Anterior cingulate cortex
9	5	0.63	Frontoparietal	Superior, middle, and inferior frontal gyri, Inferior parietal gyrus, Angular gyrus
10	13, 19	0.52, 0.46	Frontoparietal	Inferior frontal gyrus, Angular gyrus

*Cross correlation values and corresponding brain regions of ICs are reported*.

### Connectivity Differences

We adopted two-way ANOVA approach to identify brain networks that show significant interaction effects of age and symptom. One IC involved in the frontoparietal network (IC 5 and RSN 9) demonstrated significant [*F*(1,108) = 7.6047, *p* = 0.0068] interaction effects (**Table 3**). The identified IC covered bilateral superior, middle, and inferior frontal gyri, inferior parietal gyrus, and angular gyrus.

**Table 3:** Two-way ANOVA results of all ICs.

ICs	RSNs	Network	DOF	*F*-value	*p*-value
3	1	Visual	1	2.0622	0.1539
29	2	Visual	1	2.5908	0.1104
12	3	Visual	1	1.0103	0.3171
4	4	Default mode	1	0.0381	0.8457
6	4	Default mode	1	1.3595	0.2462
14	6	Sensorimotor	1	0.1797	0.6725
8	7	Auditory	1	0.2283	0.6338
9	8	Executive control	1	1.2503	0.2660
***5***	***9***	***Frontoparietal***	***1***	***7.6047***	***0.0068***
13	10	Frontoparietal	1	0.0115	0.9149
19	10	Frontoparietal	1	1.0749	0.3022

*DOF, degree of freedom.*

*Independent components with significant interaction effects are bolded and italicized*.

### Correlation with IQ

**FIGURE 2:**
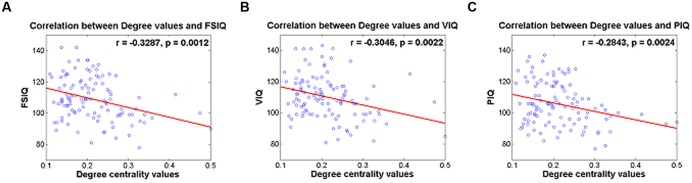
**(A)** Correlation between degree centrality values of IC 5 and FSIQ, **(B)** correlation between degree centrality values of IC 5 and VIQ, **(C)** correlation between degree centrality values of IC 5 and PIQ.

Degree values of the identified IC were correlated with IQ scores and reported in **Table 4**. IC 5 (RSN 9) demonstrated significant correlation with FSIQ, VIQ and PIQ (*r* = -0.3287 and *p* = 0.0012; *r* = -0.3046 and *p* = 0.0022; *r* = -0.2843 and *p* = 0.0024, respectively) (**Table 4**; **Figures 2A**–**C**). Correlation between degree values of identified ICs and IQ scores of three additional analysis sets were reported in Supplementary Table S4 and Supplementary Figure S1.

**Table 4:** Correlation between degree values of the identified IC and IQ scores.

ICs (RSNs)	FSIQ		VIQ		PIQ	
	*r*-value	*p*-value, corrected	*r*-value	*p*-value, corrected	*r*-value	*p*-value, corrected
***5 (9)***	***-0.3287***	***0.0012***	***-0.3046***	***0.0022***	***-0.2843***	***0.0024***

*ICs, independent components; RSNs, resting state networks; IQ, intelligence quotient; FSIQ, full scale intelligence quotient; VIQ, verbal intelligence quotient; PIQ, performance intelligence quotient*.

*Significant results (*p* < 0.05, corrected) are bolded and italicized*.

### IQ Prediction

**FIGURE 3:**
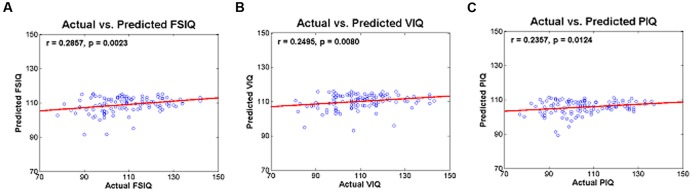
**Actual and predicted (A)** FSIQ using IC 5, **(B)** VIQ using IC 5, and **(C)** PIQ using IC 5.

Degree values of the identified IC were used to predict IQ scores in a leave-one-subject-out fashion. Degree values of the identified IC were used as regressors in a regression framework. Quality of prediction was assessed with *r*- and *p*-values, and RMS and percent-error between actual and predicted IQ scores were computed (**Table 5**). The actual and predicted FSIQ using degree values of IC 5 yielded significant results (*r* = 0.2857 and *p* = 0.0023) with an RMS error of 12.99 and a percent error of 9.81% (**Table 5**; **Figure 3A**). The actual and predicted VIQ using degree values of IC 5 yielded significant results (*r* = 0.2495 and *p* = 0.0080) with an RMS error of 13.30 and a percent error of 9.52% (**Table 5**; **Figure 3B**). The actual and predicted PIQ using degree values of IC 5 yielded significant results (*r* = 0.2357; *p* = 0.0124) with an RMS error of 13.38 and a percent error of 10.59% (**Table 5**; **Figure 3C**). IQ prediction results of three additional analysis sets were reported in Supplementary Table S5 and Figure S2.

**Table 5:** Prediction of IQ scores using degree values of the identified IC.

IQ	Information	IC 5 (RSN 9)
FSIQ	*r*-value	***0.2857***
	*p*-value	***0.0023***
	RMS error	***12.99***
	Percent error [%]	***9.81***
VIQ	*r*-value	***0.2495***
	*p*-value	***0.0080***
	RMS error	***13.30***
	Percent error [%]	***9.52***
PIQ	*r*-value	***0.2357***
	*p*-value	***0.0124***
	RMS error	***13.38***
	Percent error [%]	***10.59***

*IC, independent component; RSN, resting state network; IQ, intelligence quotient; FSIQ, full scale intelligence quotient; VIQ, verbal intelligence quotient; PIQ, performance intelligence quotient; RMS, root mean squared*.

*Significant results (r > 0 and p < 0.05) are bolded and italicized*.

## Discussion

The cingulo-fronto-parietal (CFP) network is highly related to ADHD symptoms, as it regulates attention, working memory, executive control, and response inhibitory control (Bush et al., 2005; Schneider et al., 2006; Bush, 2010; Hoekzema et al., 2014). Many ADHD studies have discovered abnormal function in the CFP network (Bush et al., 2005; Schneider et al., 2006; Bush, 2010; Oldehinkel et al., 2013; Hoekzema et al., 2014). The cingulum portion of the CFP network covers the anterior cingulate gyrus, and it is involved in IC 9 (RSN 8), the executive control network. The IC 9 (RSN 8) did not show significant interaction effects of age and symptom in our results. Only IC 5 (RSN 9), the frontoparietal network, showed significant interaction effects. The main objective of this study was to find brain network differences of ADHD patients between age groups. Our results indicated that connectivity in the frontoparietal network, not in the anterior cingulate gyrus, could explain differences between child and adolescent ADHD patients. The frontoparietal network is highly related to hyperactivity and impulsivity (Schneider et al., 2006; Bush, 2010; Oldehinkel et al., 2013) and thus altered connectivity in the frontoparietal network could explain behavioral differences in child and adolescent ADHD patients. Hence, our results corroborate those of existing studies (Schneider et al., 2006; Oldehinkel et al., 2013). IQ is known to be a significant predictor of ADHD symptoms in adolescence and early adulthood (Cheung et al., 2015). ADHD symptoms showed differential improvements in adolescents according to IQ scores in childhood (Cheung et al., 2015). The frontoparietal network plays an important role in ADHD age-related studies, as demonstrated by a previous study and our results (Li et al., 2014). We demonstrated that all IQ (FSIQ, VIQ, and PIQ) scores are highly correlated with the frontoparietal network measures, as indicated by correlation and prediction analysis. Correlation between degree centrality values of the frontoparietal network and IQ scores showed negative relationships. Degree centrality of a region measures local brain network property and thus degree centrality does not necessarily have to be correlated with clinical score such as IQ. A region with high degree centrality could be considered as an important hub node, but it does not imply positive correlation between centrality values and IQ scores. Thus, negative correlation between degree centrality of the frontoparietal network and IQ is feasible. Our results were consistent with those of a previous study (Kebir et al., 2009). IQ scores are highly associated with executive functions including attentional and inhibitory control related to ADHD symptoms (Rommelse et al., 2008; Brown et al., 2009; Kebir et al., 2009). This could explain the high degree of correlation between degree centrality values and IQ scores of ADHD patients. Our results can therefore be considered reinforcing, as neuroimaging analyses were closely linked with IQ, a known predictor of ADHD symptoms.

We randomly removed a few patients to have matched number of subjects in four comparison groups. We repeated the random removal process three more times and obtained three additional sets of four comparison groups. All four sets of data were analyzed to check the reproducibility of our findings. Demographic data were similar between the main data set and the additional data sets (**Table 1**; Supplementary Table S1). Group-ICA was performed on all four data sets and functionally interpretable ICs were reported in Supplementary Table S2. All four analyses showed significant (*p* < 0.05) interaction effects of age and symptom in the frontoparietal network and the visual network showed significant interaction effects only in the third additional set (Supplementary Table S3). Visual cortex plays an important role in child and adolescent ADHD patients (Mazaheri et al., 2010; Kroger et al., 2014). Previous studies reported altered activation in occipital regions (Kroger et al., 2014) and disconnection between frontal and occipital cortex (Mazaheri et al., 2010) in child and adolescent ADHD patients. The correlation between degree centrality values of the frontoparietal network and IQ scores were reported in Supplementary Table S4. All IQ scores were significantly (*p* < 0.05) correlated with degree values of the frontoparietal network in the first additional set. Only PIQ showed significant (*p* < 0.05) correlation in the second additional set, and VIQ and PIQ showed significant (*p* < 0.05) correlation in the third additional set. FSIQ and VIQ in the second additional set and FSIQ in the third additional set did not show significant (*p* > 0.05) correlation but the *p*-values were close to 0.05 (*p* = 0.0691; *p* = 0.0585; *p* = 0.0559, respectively). These results imply that those IQ scores were not significantly correlated at 0.05 level but still moderately correlated (e.g., *p*-value between 0.5 and 0.6) to degree values of the frontoparietal network. The IQ prediction results were reported in Supplementary Table S5. The percent error values were approximately 10% for all additional data sets. In sum, we confirmed that our results stayed consistent even if we removed different set of random subjects.

Child and adolescent ADHD patients are known to show different behavior symptoms, particularly hyperactivity and impulsivity (Wehmeier et al., 2010; Wolraich et al., 2011). Adolescent ADHD patients rarely exhibit hyperactive and impulsive behaviors compared to child ADHD patients (Hurtig et al., 2007; Wehmeier et al., 2010; Wolraich et al., 2011). A previous study reported that ADHD combined type patients were the most common in childhood (43%), while ADHD inattentive type patients were the most common in adolescence (64%) (Hurtig et al., 2007). Changes in environmental situations such as maturation, desire to be independent from parents, and spending more time away from home might be explanations for behavioral differences between child and adolescent ADHD patients (Wehmeier et al., 2010). Treatment options should consider these behavioral differences, as indiscriminate treatments might negatively affect ADHD patients resulting in poor academic performance and high risk of substance abuse (Barkley et al., 1996; Barnard et al., 2010; Wehmeier et al., 2010; Wolraich et al., 2011). Adolescent ADHD patients not treated in childhood are more likely to be suspended from school, be socially excluded, get in car accidents, and have comorbid disorders such as substance abuse, dependence, and mood disorders than those who receive treatment in childhood (Barkley et al., 1996). In sum, comprehensive understanding of ADHD might require understanding of age-related behavioral differences. Our study provides insight to behavioral differences between child and adolescent ADHD patients via state of the art connectivity analysis.

Our study has some limitations. First, there were no significant differences in ADHD hyperactive/impulsive scores between child and adolescent ADHD patients although the behavioral differences of hyperactivity and impulsivity between child and adolescent ADHD patients are well established. We were limited by the available neuroimaging data of ADHD-200 database and future studies with more samples could solve this issue. Also, our result showed that there are significant relationships between degree centrality values of the frontoparietal network and IQ scores and further studies with more samples are needed to fully interpret correlation between IQ scores and centrality values. Second, we only used degree centrality as a connectivity measure. There are several other measures such as betweenness, eigenvector, and closeness centrality (Rubinov and Sporns, 2010). All centrality measures quantify the importance of a given node, but there is no single ideal measure for a plethora of research questions (Rubinov and Sporns, 2010; Zuo et al., 2012; dos santos Siqueira et al., 2014). Other centrality measures might have improved sensitivity for assessing ADHD related brain networks. Finally, we only used rs-fMRI data. A multi-modality study incorporating many neuroimaging modalities might provide complementary information to better assess ADHD brains.

We identified brain networks that showed significant interaction effects of age (child and adolescent) and symptom (ADHD and NC) using group ICA and weighted degree values. The frontoparietal network showed significant interaction effects (*p* = 0.0068) and the degree values of the frontoparietal network demonstrated high correlation with IQ scores (average *r* = 0.31). Furthermore, actual and predicted IQ scores yielded significant results, with an approximate error of 10%. Our study suggests a possible statistical link among behavioral symptom differences (i.e., hyperactivity and impulsivity) between child and adolescent ADHD patients and brain networks, and our study might provide potential imaging biomarkers for future ADHD and intelligence studies.

## Author Contributions

B-yP and HP wrote the manuscript and JH and S-HL aided the experiments. HP is the guarantor of this work and, as such, had full access to all the data in the study and takes responsibility for the integrity of the data and the accuracy of the data analysis.

## Conflict of Interest Statement

The authors declare that the research was conducted in the absence of any commercial or financial relationships that could be construed as a potential conflict of interest.
