# Supplementary material for: Functional Connectivity of Child and Adolescent Attention Deficit Hyperactivity Disorder Patients: Correlation with IQ
Source: Front Hum Neurosci. 2016 Nov 9;10:565. doi: 10.3389/fnhum.2016.00565 (PMC5101198; doi:10.3389/fnhum.2016.00565)
Supplement: Supplementary file 1 [file Data_Sheet_1.DOCX]

Supplementary Material

Functional connectivity of child and adolescent attention deficit hyperactivity disorder patients: correlation with IQ

Bo-yong Park, Jisu Hong, Seung-Hak Lee and Hyunjin Park*

*** Correspondence:** Hyunjin Park: hyunjinp@skku.edu

# Supplementary Figures and Tables

## Supplementary Figures


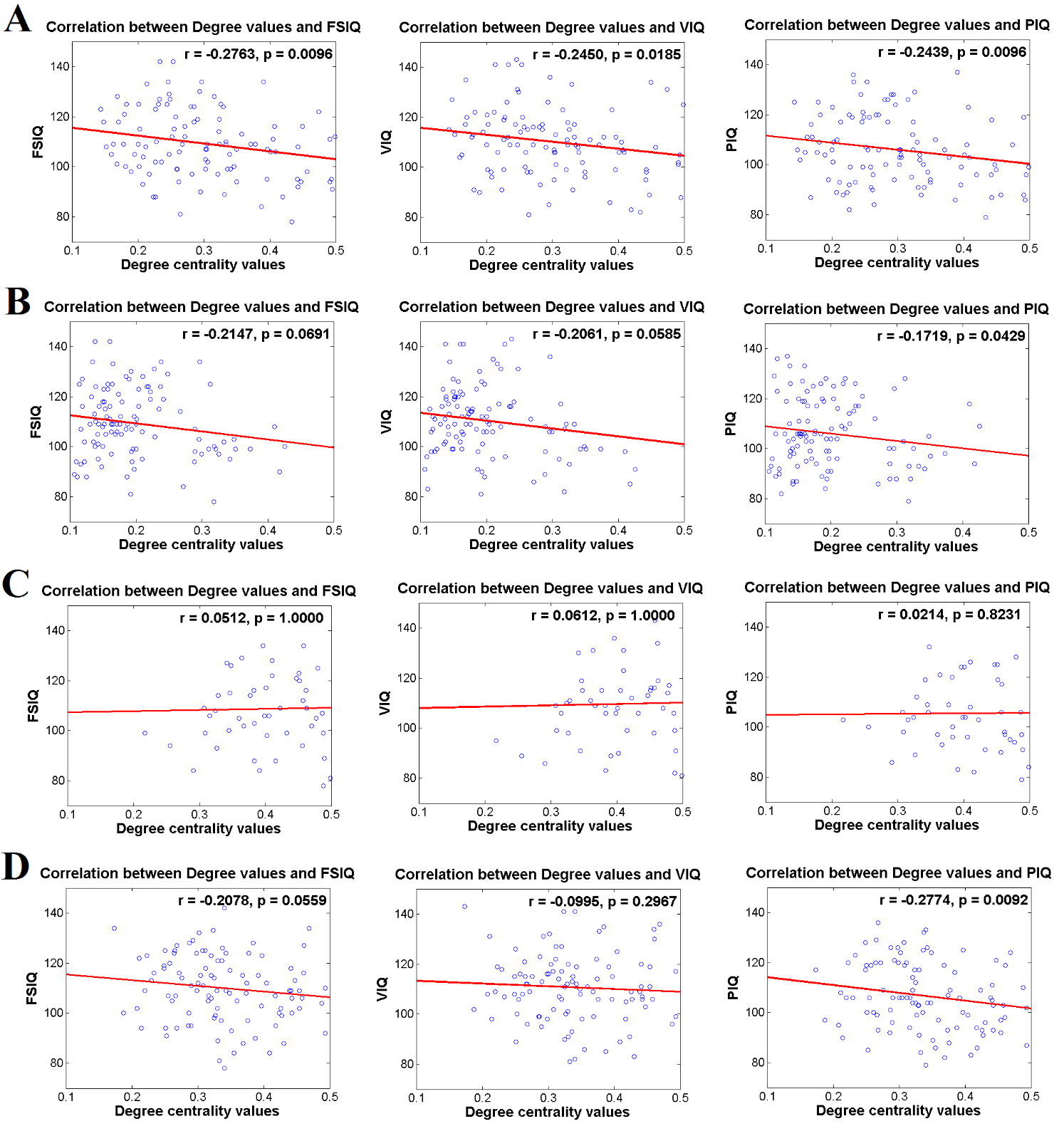


**Supplementary Figure 1.** (A) Correlation between degree centrality values of IC 15 and IQ scores of set 1, (B) correlation between degree centrality values of IC 7 and IQ scores of set 2, (C) correlation between degree centrality values of IC 26 and IQ scores of set 3, and (D) correlation between degree centrality values of IC 2 and IQ scores of set 3.

**
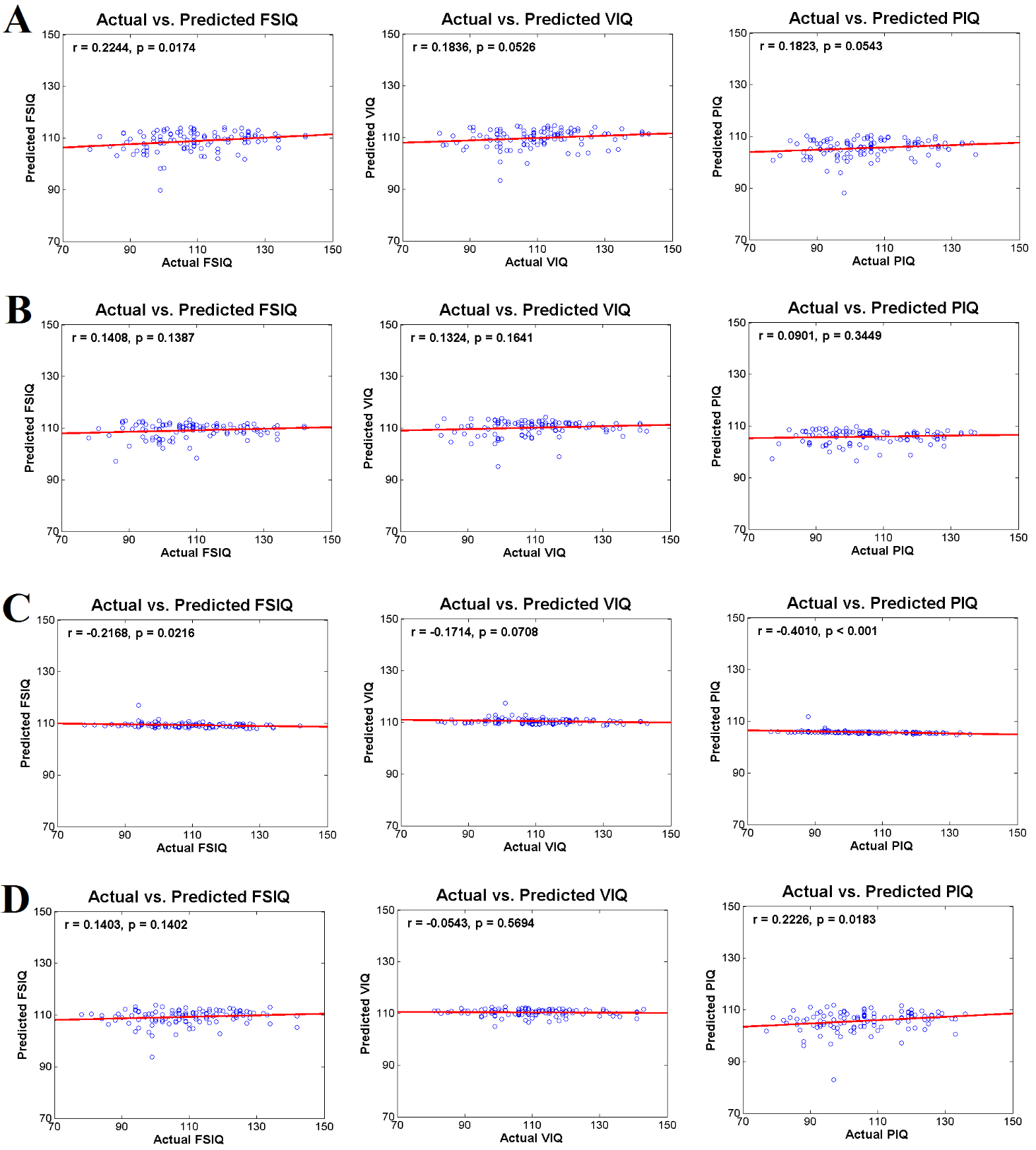
**

**Supplementary Figure 2.** Actual and predicted IQ scores using (A) IC 15 of set 1, (B) IC 7 of set 2, (C) IC 26 of set 3 and (D) IC 2 of set 3.

## Supplementary Tables

**Supplementary Table 1.** Demographic data of child and adolescent subjects in the ADHD and NC groups with different data sets (means and standard deviations are reported).

| ***Set 1*** | | | |
| --- | --- | --- | --- |
| **ADHD Group** | **CHILD (*n* = 28)** | **ADOLESCENT (*n* = 28)** | ***p*-value** |
| Gender (Male : Female) | 22:6 | 22:6 | * 1 |
| Age (years) | 8.59 (0.76) | 12.22 (1.79) | < 0.001 |
| IA score | 71.82 (8.74) | 72.82 (8.65) | 0.6687 |
| HI score | 68.21 (11.89) | 72.11 (11.98) | 0.2276 |
| C score | 72.14 (8.10) | 75.04 (9.38) | 0.2222 |
| Subtype (IA : HI : C) | 7:0:21 | 6:1:21 | *0.8084 |
| FSIQ | 110.11 (13.70) | 104.07 (13.58) | 0.1036 |
| VIQ | 110.79 (12.70) | 104.71 (12.76) | 0.0799 |
| PIQ | 106.54 (14.99) | 102.21 (14.35) | 0.2754 |
| **NC Group** | **CHILD (*n* = 28)** | **ADOLESCENT (*n* = 28)** | ***p*-value** |
| Gender (Male : Female) | 14:14 | 14:14 | * 1 |
| Age (years) | 8.50 (0.78) | 13.74 (2.37) | < 0.001 |
| FSIQ | 109.04 (12.67) | 111.86 (13.57) | 0.4248 |
| VIQ | 111.46 (14.74) | 112.07 (13.48) | 0.8728 |
| PIQ | 104.61 (12.58) | 108.75 (12.70) | 0.2254 |

| ***Set 2*** | | | |
| --- | --- | --- | --- |
| **ADHD Group** | **CHILD (*n* = 28)** | **ADOLESCENT (*n* = 28)** | ***p*-value** |
| Gender (Male : Female) | 22:6 | 22:6 | * 1 |
| Age (years) | 8.59 (0.76) | 12.56 (1.97) | < 0.001 |
| IA score | 71.82 (8.74) | 71.32 (8.94) | 0.8332 |
| HI score | 68.21 (11.89) | 70.50 (11.82) | 0.4738 |
| C score | 72.14 (8.10) | 73.61 (9.65) | 0.5411 |
| Subtype (IA : HI : C) | 7:0:21 | 7:1:20 | *0.9372 |
| FSIQ | 110.11 (13.70) | 105.32 (14.35) | 0.2073 |
| VIQ | 110.79 (12.70) | 106.25 (14.12) | 0.2117 |
| PIQ | 106.54 (14.99) | 103.11 (14.64) | 0.3905 |
| **NC Group** | **CHILD (*n* = 28)** | **ADOLESCENT (*n* = 28)** | ***p*-value** |
| Gender (Male : Female) | 14:14 | 14:14 | * 1 |
| Age (years) | 8.50 (0.78) | 13.77 (2.34) | < 0.001 |
| FSIQ | 109.04 (12.67) | 111.79 (13.50) | 0.4354 |
| VIQ | 111.46 (14.74) | 112.00 (13.43) | 0.8875 |
| PIQ | 104.61 (12.58) | 109.00 (13.00) | 0.2042 |

| ***Set 3*** | | | |
| --- | --- | --- | --- |
| **ADHD Group** | **CHILD (*n* = 28)** | **ADOLESCENT (*n* = 28)** | ***p*-value** |
| Gender (Male : Female) | 22:6 | 22:6 | * 1 |
| Age (years) | 8.59 (0.76) | 12.63 (2.22) | < 0.001 |
| IA score | 71.82 (8.74) | 72.46 (7.43) | 0.7680 |
| HI score | 68.21 (11.89) | 72.61 (11.72) | 0.1695 |
| C score | 72.14 (8.10) | 74.32 (8.77) | 0.3385 |
| Subtype (IA : HI : C) | 7:0:21 | 8:0:20 | *0.7628 |
| FSIQ | 110.11 (13.70) | 103.29 (13.61) | 0.0670 |
| VIQ | 110.79 (12.70) | 105.68 (13.46) | 0.1500 |
| PIQ | 106.54 (14.99) | 99.82 (13.11) | 0.0800 |
| **NC Group** | **CHILD (*n* = 28)** | **ADOLESCENT (*n* = 28)** | ***p*-value** |
| Gender (Male : Female) | 14:14 | 14:14 | * 1 |
| Age (years) | 8.50 (0.78) | 13.53 (2.13) | < 0.001 |
| FSIQ | 109.04 (12.67) | 114.57 (12.04) | 0.0996 |
| VIQ | 111.46 (14.74) | 113.54 (12.34) | 0.5709 |
| PIQ | 104.61 (12.58) | 111.93 (11.90) | 0.0294 |

* Chi-squared test.

ADHD, attention deficit hyperactivity disorder; NC, normal controls; IA, inattentive; HI, hyperactive/impulsive; C, combined; FSIQ, full scale intelligence quotient; VIQ, verbal intelligence quotient; PIQ, performance intelligence quotient

**Supplementary Table 2.** Functionally interpretable ICs and RSNs of different data sets. Cross correlation values and corresponding regions of ICs are reported.

| ***Set 1*** | | | | |
| --- | --- | --- | --- | --- |
| **RSNs** | **ICs** | ***r*-value** | **Network** | **Region** |
| 1 | 2 | 0.81 | Visual | Calcarine  Cuneus  Lingual gyrus |
| 2 | 27 | 0.79 | Visual | Inferior occipital gyrus |
| 3 | 8, 18 | 0.57, 0.43 | Visual | Superior, middle, and inferior occipital gyri |
| 4 | 6 | 0.63 | Default Mode | Posterior cingulate cortex  Cuneus |
| 5 | - | - | Cerebellum | - |
| 6 | 9, 16 | 0.41, 0.47 | Sensorimotor | Superior and inferior parietal gyri  Postcentral gyrus  Paracentral lobule |
| 7 | 4 | 0.64 | Auditory | Rolandic operculum  Insula  Putamen  Pallidum  Heschl’s gyrus |
| 8 | 7, 17 | 0.44, 0.63 | Executive Control | Medial orbitofrontal gyrus  Anterior cingulate cortex  Amygdala  Putamen  Pallidum |
| 9 | 5 | 0.62 | Frontoparietal | Middle and inferior frontal gyri  Inferior parietal gyrus  Angular gyrus |
| 10 | 14, 15 | 0.56, 0.43 | Frontoparietal | Inferior frontal gyrus  Inferior parietal gyrus  Angular gyrus |

| ***Set 2*** | | | | |
| --- | --- | --- | --- | --- |
| **RSNs** | **ICs** | ***r*-value** | **Network** | **Region** |
| 1 | 4 | 0.81 | Visual | Calcarine  Cuneus  Lingual gyrus  Superior occipital gyrus |
| 2 | 17 | 0.78 | Visual | Inferior occipital gyrus |
| 3 | 6 | 0.48 | Visual | Superior, middle, and inferior occipital gyri  Superior parietal lobule |
| 4 | 3, 8 | 0.52, 0.64 | Default Mode | Medial orbitofrontal gyrus  Posterior cingulate cortex  Cuneus |
| 5 | - | - | Cerebellum | - |
| 6 | 13 | 0.47 | Sensorimotor | Paracentral lobule |
| 7 | 5 | 0.62 | Auditory | Rolandic operculum  Insula  Amygdala  Putamen  Pallidum  Thalamus  Heschl’s gyrus  Superior temporal gyrus |
| 8 | 9 | 0.71 | Executive Control | Superior medial frontal gyrus  Anterior cingulate cortex  Caudate |
| 9 | 7 | 0.61 | Frontoparietal | Superior, middle and inferior frontal gyri  Inferior parietal gyrus  Angular gyrus |
| 10 | 11 | 0.55 | Frontoparietal | Angular gyrus |

| ***Set 3*** | | | | |
| --- | --- | --- | --- | --- |
| **RSNs** | **ICs** | ***r*-value** | **Network** | **Region** |
| 1 | 3 | 0.80 | Visual | Calcarine  Cuneus  Lingual gyrus |
| 2 | 26 | 0.79 | Visual | Inferior occipital gyrus |
| 3 | 10 | 0.51 | Visual | Superior, middle, and inferior occipital gyri  Superior parietal lobule |
| 4 | 4, 7 | 0.49, 0.64 | Default Mode | Medial orbitofrontal gyrus  Posterior cingulate cortex  Cuneus |
| 5 | - | - | Cerebellum | - |
| 6 | 16 | 0.47 | Sensorimotor | Paracentral lobule |
| 7 | 6 | 0.66 | Auditory | Rolandic operculum  Insula  Putamen  Pallidum  Heschl’s gyrus  Superior temporal gyrus |
| 8 | 8 | 0.72 | Executive Control | Superior medial frontal gyrus  Anterior cingulate cortex |
| 9 | 2 | 0.61 | Frontoparietal | Superior, middle and inferior frontal gyri  Inferior parietal gyrus  Angular gyrus |
| 10 | 12 | 0.54 | Frontoparietal | Angular gyrus |

ICs, independent components; RSNs, resting state networks

**Supplementary Table 3.** Two-way ANOVA results of all ICs of different data sets. ICs with significant interaction effects are bolded and italicized.

| ***Set 1*** | | | | | |
| --- | --- | --- | --- | --- | --- |
| **ICs** | **RSNs** | **Network** | **DOF** | ***F*-value** | ***p*-value** |
| 2 | 1 | Visual | 1 | 1.0105 | 0.3170 |
| 27 | 2 | Visual | 1 | 2.0866 | 0.1515 |
| 8 | 3 | Visual | 1 | 0.7857 | 0.3774 |
| 18 | 3 | Visual | 1 | 0.4435 | 0.5069 |
| 6 | 4 | Default mode | 1 | 0.0392 | 0.8433 |
| 9 | 6 | Sensorimotor | 1 | 1.9917 | 0.1610 |
| 16 | 6 | Sensorimotor | 1 | 0.1033 | 0.7485 |
| 4 | 7 | Auditory | 1 | 0.4618 | 0.4983 |
| 7 | 8 | Executive control | 1 | 0.5200 | 0.4724 |
| 17 | 8 | Executive control | 1 | 0.0722 | 0.7887 |
| 5 | 9 | Frontoparietal | 1 | 2.5940 | 0.1102 |
| 14 | 10 | Frontoparietal | 1 | 0.0001 | 0.9908 |
| ***15*** | ***10*** | ***Frontoparietal*** | ***1*** | ***4.3108*** | ***0.0402*** |

| ***Set 2*** | | | | | |
| --- | --- | --- | --- | --- | --- |
| **ICs** | **RSNs** | **Network** | **DOF** | ***F*-value** | ***p*-value** |
| 4 | 1 | Visual | 1 | 1.7302 | 0.1912 |
| 17 | 2 | Visual | 1 | 2.1207 | 0.1482 |
| 6 | 3 | Visual | 1 | 0.9751 | 0.3256 |
| 3 | 4 | Default mode | 1 | 0.4949 | 0.4833 |
| 8 | 4 | Default mode | 1 | 0.1120 | 0.7386 |
| 13 | 6 | Sensorimotor | 1 | 0.1007 | 0.7516 |
| 5 | 7 | Auditory | 1 | 0.0093 | 0.9235 |
| 9 | 8 | Executive control | 1 | 1.2681 | 0.2626 |
| ***7*** | ***9*** | ***Frontoparietal*** | ***1*** | ***7.5479*** | ***0.0070*** |
| 11 | 10 | Frontoparietal | 1 | 0.8535 | 0.3576 |

| ***Set 3*** | | | | | |
| --- | --- | --- | --- | --- | --- |
| **ICs** | **RSNs** | **Network** | **DOF** | ***F*-value** | ***p*-value** |
| 3 | 1 | Visual | 1 | 2.8606 | 0.0937 |
| ***26*** | ***2*** | ***Visual*** | ***1*** | ***5.9428*** | ***0.0164*** |
| 10 | 3 | Visual | 1 | 2.6735 | 0.1049 |
| 4 | 4 | Default mode | 1 | 0.0080 | 0.9291 |
| 7 | 4 | Default mode | 1 | 0.0001 | 0.9915 |
| 16 | 6 | Sensorimotor | 1 | 0.5842 | 0.4463 |
| 6 | 7 | Auditory | 1 | 1.0955 | 0.2976 |
| 8 | 8 | Executive control | 1 | 0.3137 | 0.5766 |
| ***9*** | ***9*** | ***Frontoparietal*** | ***1*** | ***6.1655*** | ***0.0146*** |
| 12 | 10 | Frontoparietal | 1 | 0.0020 | 0.9640 |

ICs, independent components; RSNs, resting state networks; ADHD, attention deficit hyperactivity disorder; NC, normal controls

**Supplementary Table 4.** Correlation between degree values of identified ICs and IQ scores of different data sets. Significant results (*p* < 0.05, corrected) are bolded and italicized.

| ***Set 1*** | | | | | | |
| --- | --- | --- | --- | --- | --- | --- |
| **ICs (RSNs)** | **FSIQ** | | **VIQ** | | **PIQ** | |
|  | ***r*-value** | ***p*-value, corrected** | ***r*-value** | ***p*-value, corrected** | ***r*-value** | ***p*-value, corrected** |
| 15 (10) | ***-0.2763*** | ***0.0096*** | ***-0.2450*** | ***0.0185*** | ***-0.2439*** | ***0.0096*** |
| ***Set 2*** | | | | | | |
| **ICs (RSNs)** | **FSIQ** | | **VIQ** | | **PIQ** | |
|  | ***r*-value** | ***p*-value, corrected** | ***r*-value** | ***p*-value, corrected** | ***r*-value** | ***p*-value, corrected** |
| 7 (9) | -0.2147 | 0.0691 | -0.0261 | 0.0585 | ***-0.1719*** | ***0.0367*** |
| ***Set 3*** | | | | | | |
| **ICs (RSNs)** | **FSIQ** | | **VIQ** | | **PIQ** | |
|  | ***r*-value** | ***p*-value, corrected** | ***r*-value** | ***p*-value, corrected** | ***r*-value** | ***p*-value, corrected** |
| 26 (2) | 0.0512 | 1.0000 | 0.0612 | 1.0000 | 0.0214 | 0.8231 |
| 2 (9) | -0.2078 | 0.0559 | ***-0.0995*** | ***0.2967*** | ***-0.2774*** | ***0.0092*** |

ICs, independent components; RSNs, resting state networks; IQ, intelligence quotient; FSIQ, full scale intelligence quotient; VIQ, verbal intelligence quotient; PIQ, performance intelligence quotient

**Supplementary Table 5.** Prediction of IQ scores using degree values of identified ICs of different data sets. Significant results (*r* > 0 and *p* < 0.05) are bolded and italicized.

| ***Set 1*** | | | | | |
| --- | --- | --- | --- | --- | --- |
| **IQ** | | **Information** | | **IC 15 (RSN 10)** | |
| FSIQ | | *r*-value | | ***0.2244*** | |
|  |  | *p*-value | | ***0.0174*** | |
|  |  | RMS error | | ***13.14*** | |
|  |  | Percent error [%] | | ***10.08*** | |
| VIQ | | *r*-value | | 0.1836 | |
|  |  | *p*-value | | 0.0526 | |
|  |  | RMS error | | 13.33 | |
|  |  | Percent error [%] | | 9.47 | |
| PIQ | | *r*-value | | 0.1823 | |
|  |  | *p*-value | | 0.0543 | |
|  |  | RMS error | | 13.47 | |
|  |  | Percent error [%] | | 10.40 | |
| ***Set 2*** | | | | | |
| **IQ** | | **Information** | | **IC 7 (RSN 9)** | |
| FSIQ | | *r*-value | | 0.1408 | |
|  |  | *p*-value | | 0.1387 | |
|  |  | RMS error | | 13.43 | |
|  |  | Percent error [%] | | 10.10 | |
| VIQ | | *r*-value | | 0.1324 | |
|  |  | *p*-value | | 0.1641 | |
|  |  | RMS error | | 13.63 | |
|  |  | Percent error [%] | | 9.76 | |
| PIQ | | *r*-value | | 0.0901 | |
|  |  | *p*-value | | 0.3449 | |
|  |  | RMS error | | 13.74 | |
|  |  | Percent error [%] | | 10.98 | |
| ***Set 3*** | | | | | |
| **IQ** | **Information** | | **IC 26 (RSN 2)** | | **IC 2 (RSN 9)** |
| FSIQ | *r*-value | | -0.2168 | | 0.1403 |
|  | *p*-value | | 0.0216 | | 0.1402 |
|  | RMS error | | 13.65 | | 13.31 |
|  | Percent error [%] | | 10.41 | | 10.05 |
| VIQ | *r*-value | | -0.1714 | | -0.0543 |
|  | *p*-value | | 0.0708 | | 0.5694 |
|  | RMS error | | 13.63 | | 13.55 |
|  | Percent error [%] | | 9.90 | | 9.78 |
| PIQ | *r*-value | | -0.4010 | | ***0.2226*** |
|  | *p*-value | | < 0.001 | | ***0.0183*** |
|  | RMS error | | 13.63 | | ***13.35*** |
|  | Percent error [%] | | 9.90 | | ***10.59*** |

IC, independent component; RSN, resting state network; IQ, intelligence quotient; FSIQ, full scale intelligence quotient; VIQ, verbal intelligence quotient; PIQ, performance intelligence quotient; RMS, root mean squared
